# Irisin as a Multifunctional Protein: Implications for Health and Certain Diseases

**DOI:** 10.3390/medicina55080485

**Published:** 2019-08-15

**Authors:** Paulina Korta, Ewa Pocheć, Agnieszka Mazur-Biały

**Affiliations:** 1 Department of Glycoconjugate Biochemistry, Institute of Zoology and Biomedical Research, Faculty of Biology, Jagiellonian University, Gronostajowa 9, 30-387 Krakow, Poland; 2 Department of Ergonomics and Exercise Physiology, Faculty of Health Sciences, Jagiellonian University, Medical College, Grzegorzecka 20, 31-531 Krakow, Poland

**Keywords:** irisin, FNDC5, *N*-glycosylation, physical activity, obesity, inflammation, cancers

## Abstract

Sedentary life style is considered to be an independent risk factor for many disorders, including development of type 2 diabetes, obesity, immune dysfunction, asthma, and neurological or coronary heart disease. Irisin is released from myocytes during physical activity, and acts as a link between muscles and other tissues and organs. This myokine is produced as a result of proteolytic cleavage of FNDC5 protein present in the membrane of myocytes. Secretion of irisin is regulated by *N*-linked oligosaccharides attached to the protein molecule. The two *N*-glycan molecules, which constitute a significant part of the irisin glycoprotein, regulate the browning of adipocytes, which is the most important function of irisin. A receptor specific for irisin has still not been discovered. In some tissues irisin probably acts via integrins, which are widely expressed transmembrane receptors. Many studies have confirmed the multifunctional role of irisin and the beneficial effects of this molecule on body homeostasis. Irisin reduces systemic inflammation, maintains the balance between resorption and bone formation, and modulates metabolic processes and the functioning of the nervous system. It suppresses the expression and release of pro-inflammatory cytokines in obese individuals and attenuates inflammation in adipose tissue. The impact of irisin on cancer cell proliferation, migration, and invasion has also been demonstrated in numerous studies, which proves its role in carcinogenesis. Owing to these pleiotropic and beneficial properties, irisin may be a potential option to prevent and treat civilization-related diseases which are, nowadays, considered to be the major health problems in Western societies.

## 1. Introduction

Skeletal muscle is the largest organ in the human body [1]. During or immediately after physical exercise myocytes secrete molecules called myokines, mainly chemokines and cytokines. Myokines regulate a variety of metabolic processes in various tissues and organs, such as liver, bones, brain, or fat tissue through endocrine, paracrine, or autocrine signaling pathways. The major myokines include interleukin 6 (IL-6), monocyte chemotactic protein 1 (MCP1), insulin-like growth factor-1 (IGF-1), and myostatin [2,3]. In 2012, Boström et al. reported the discovery of a new molecule that is secreted by myocytes. That molecule was able to induce changes in adipose tissue and activate thermogenesis [4]. Moreover, that molecule was proposed to act as a link between the muscles and other tissues of the body; the newly discovered protein has been called “irisin,” derived from the name of the Greek goddess Iris [5]. Since then, irisin has been the subject of extensive research, which enabled the gaining of insight into its pleiotropic properties.

## 2. Structure and *N*-Glycosylation of Irisin

**Figure 1:**
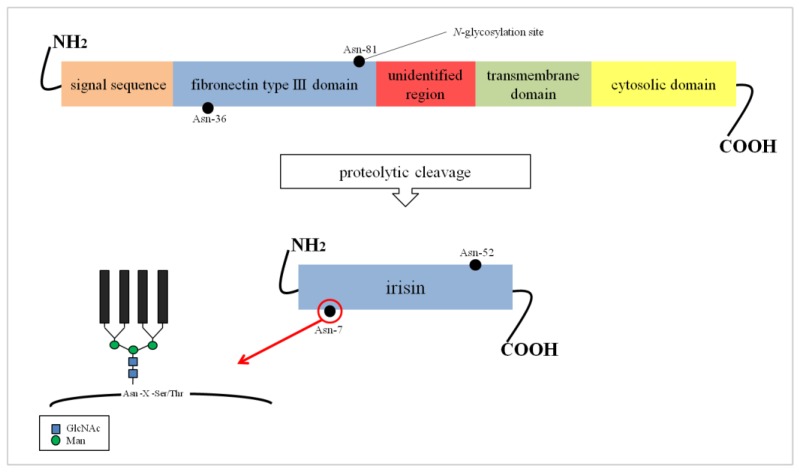
FNDC5 structure and formation of irisin. The potential *N*-glycosylation sites are marked as black dots. Asn, asparagine; GlcNAc, N-acetylglucosamine; Man, mannose; Ser, serine; Thr, threonine; X, any amino acid except proline.

Irisin is a fragment of a cell membrane protein called fibronectin type III domain-containing protein 5 (FNDC5/FRCP2/PeP) [4]. The FNDC5 protein is composed of 209 amino acid (aa) residues. It contains an N-terminal signal sequence built of 29 aa, a fibronectin type III domain with 94 aa, an unidentified region consisting of 28 aa, a transmembrane domain having 19 aa, and a C-terminal part with 39 aa (Figure 1). The C-terminal fragment of FNDC5 is located in the cytoplasm, while the extracellular N-terminal potion is proteolytically cleaved to produce irisin which is ultimately released into the circulation [6,7]. Both biochemical and X-ray crystallography studies showed that irisin occurs in the form of homodimers, where a β-sheet is created between the units. This structure is stabilized not only by hydrogen bonds, but also by interactions between the side chains of adjacent subunits, in particular, between Arg-75 and Glu-79, which in turn protect the dimer ends and Trp-90/Trp-90 [8]. It was previously established that irisin is composed of 112 aa. The mass of FNDC5 proteins range from 20 to 32 kDa depending on the number and structure of oligosaccharides (glycans) attached to the protein molecule during the post-translational process of *N*-glycosylation [4,9].

Glycosylation is one of the most common post-translational modifications of proteins which occurs in the lumen of endoplasmic reticulum and the Golgi apparatus. More than half of all proteins are glycosylated, mainly cell membrane and secreted proteins. The attachment of carbohydrates, which is a multi-stage process regulated by hundreds of enzymes, leads to a great heterogeneity in the glycan structures. Oligosaccharides affect the physicochemical properties of proteins, are necessary to obtain the accurate conformation of proteins, provide protection against proteolysis, and are important for their biological functions in different metabolic processes [10]. FNDC5 is an *N*-glycosylated protein and contains oligosaccharides attached to the asparagine residue in the Asn–X–Ser/Thr sequence (where X is any amino acid except proline), via a *N*-acetylglucosamine residue (GlcNAc) [11]. Three main groups of *N*-glycan structures are linked via *N*-glycosidic bonds to Asn: High-mannose/oligomannose type, complex type and hybrid type. All *N*-glycans contain the same pentasaccharide core but differ in the composition of the side chains. The external portions of high-mannose glycans consist of 5–9 mannose (Man) residues. Complex-type oligosaccharides are characterized by a more diverse structures composed of GlcNAc, galactose (Gal), fucose (Fuc), and sialic acid (SA) residues. The hybrid-type constitutes an intermediate class of *N*-glycans where one arm is built of mannose residues and the other arm resembles the structures of complex-type glycans [12].

The glycosylation process of FNDC5/irisin is still poorly characterized. The sequence of FNDC5 contains three potential *N*-glycosylation sites and two of them, Asn-36 and Asn-81, are occupied by *N*-glycans (Figure 1). However, their structure has not been determined so far. The absence of oligosaccharides has a significant effect on the stability of the molecule. It was found that the de-*N*-glycosylated FNDC5 is more sensitive to the action of protein synthesis inhibitors compared to the glycosylated molecule. The removal of one *N*-glycosylation site by site-directed mutagenesis resulted in a significant reduction in the stability of FNDC5, with a half-life of about 7 h compared to the 12 h characteristic of the fully glycosylated protein. The de-*N*-glycosylated FNDC5 does not achieve a normal spatial conformation and is not incorporated into the cell membrane. That results in a significant decrease in irisin secretion into the blood [11,13].

**Figure 2:**
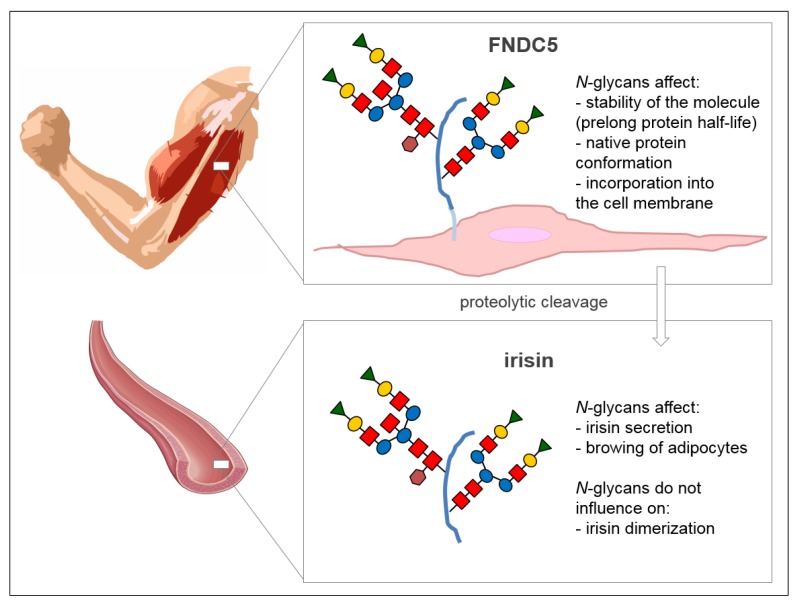
The role of *N*-glycans attached to FNDC5 and irisin proteins. Schematic structure of *N*-glycan: Red squares, *N*-acetylglucosamine; blue circles, mannose; yellow circles, galactose; green triangles, sialic acid; brown hexagons, fucose. Scheme of an artery comes from the website SMART Sevier Medical Art (https://smart.servier.com) published by Les Laboratoires Servier.

Irisin also has two *N*-glycosylation sites located at Asn-7 and Asn-52 positions [14]. Deglycosylation of irisin lowers its molecular weight to 12 kDa [15] or 15 kDa [14]. The addition of one or two sugar chains increases its mass to 22 kDa or 25 kDa, respectively. Both *N*-glycans are probably important to the primary function of irisin in the browning of adipocytes, which was evident by an up-regulation of mitochondria uncoupling protein-1 (UCP-1) expression and its transcriptional factor peroxisome proliferator-activated receptor γ (PPARγ) coactivator-1α (PGC-1α) in the presence of irisin [14]. Glycans do not affect the formation of irisin dimers (Figure 2) [8].

## 3. Occurrence, Serum Concentration, and Possible Receptors of Irisin

Irisin is an adipomyokine secreted mainly by skeletal muscles as well as subcutaneous and visceral adipose tissues. Immunohistochemical studies showed that smaller amounts of irisin are also produced by testes, liver, pancreas, brain, spleen, heart, and stomach [16,17]. Boström et al. demonstrated that the level of irisin increases in the blood after physical exercise. They observed a 65% increase in irisin concentration in mice running regularly for 3 weeks. An increase in the level of this adipomyokine by two times was also found in the blood of healthy people after 10 weeks of supervised training [4]. Irisin concentration was found to be higher in active rather than in sedentary subjects (*p* = 0.006), and its level also depends on the activities performed at residential place, because its levels were found to be significantly higher in the serum of rural individuals compared to urban inhabitants (*p* < 0.0001) [18]. Furthermore, physical exercise is known to increase the level of irisin in people with metabolic disorders [19]. However, the impact of exercise on irisin concentration in the blood is not clear. People who train regularly usually show a lower irisin serum level [20]. It is suspected that the type of physical activity plays a role, because irisin upregulation was noted after high-intensity exercise [21] and after resistance training, but not after endurance exercise [22]. Whole-body vibration training also contributes to the elevation of irisin concentration [23]. In addition to physical exercise, diet, and hormonal regulation also affect the irisin levels [24]. The pathological conditions associated with different diseases significantly influence the release of irisin into the blood circulation. Lower irisin concentrations were observed in obese individuals and patients suffering from type 2 diabetes [25], chronic renal failure [26], and prolonged hypothyroidism [27]. The concentration of irisin was found to be about 3.6 ng/mL in the blood of sedentary people and rises to 4.3 ng/mL in active subjects [15].

Till date, no specific receptor for irisin has been identified. The results of recent studies have shown that in some tissues irisin exerts its action via binding to integrins, especially the members of αv integrin family [28]. Integrins are widely expressed transmembrane receptors that bind extracellular matrix ligands (cell–matrix interactions), membrane proteins of neighboring cells (intercellular interactions), and recognize soluble ligands [29]. They are responsible for the adhesion, migration, and aggregation of cells [30]. Stable noncovalent interactions between 18 α-subunits and eight β-subunits produce 24 functionally different integrin heterodimers [29]. Kim et al. demonstrated the binding of irisin to several integrins present in the fat cells and osteocytes, including α1β1, and with the highest affinity, to α_V_β5 integrin. The treatment of osteocytes with irisin significantly increased the phosphorylation level of focal adhesion kinase (FAK), the major intracellular signal molecule responsible for integrin signaling. The use of RGD peptide which binds to αvβ5 and acts as an integrin inhibitor, suppressed any signaling response triggered by irisin. The authors suggest that the heterodimers belonging to the αv integrin family are probably the main receptors for irisin in all the tissues [28].

## 4. Pleiotropic Activity of Irisin

### 4.1. Irisin and Adipose Tissue

One of the most important functions of irisin is the capability to induce changes in adipose tissue. Based on the structure and function of fat cells, white/yellow (WAT) and brown (BAT) adipose tissues were distinguished. WAT mainly consists of mature white adipocytes with a peripherally located nucleus and a single big lipid drop. WAT functions by accumulating excess energy in the form of triglycerides, protects organs against mechanical damage, and releases adipokines which regulate various biological processes, including inflammatory reactions [31,32,33]. Thermogenesis takes place in BAT and hence is important to maintain the body temperature. BAT is morphologically different from WAT because it contains many small lipid drops, a centrally located nucleus and a large number of mitochondria. The lipids present in BAT are primarily used for oxidative phosphorylation and heat generation. UPC-1, also named thermogenin, is expressed in the mitochondrial membrane of BAT, and plays a crucial role in these processes [33,34,35]. During physical exercise, PGC-1α is activated in the skeletal muscles and regulates the transcription of FNDC5 protein. An increase in PGC-1α level is accompanied by an elevation of mitochondrial biogenesis. PGC-1α regulates gluconeogenesis and affects the biosynthesis of heme. In BAT, PGC-1α together with irisin, modulates the expression of UCP-1 and thermogenesis. As a result, the energy consumption increases and the metabolism of lipids and glucose are driven [36,37,38]. Zhang et al. showed that irisin also affects the functioning of WAT, and the effects of its actions depend on the degree of cell differentiation. The effects of irisin were evaluated on mature adipocytes and undifferentiated preadipocytes in in vitro studies. Irisin increased the expression of UCP-1 in mature fat cells leading to the conversion of the phenotype from WAT to BAT by a process named browning. That results in the formation of a third type of adipose tissue called beige adipocyte tissue. The process of browning was regulated by irisin-induced phosphorylation of mitogen-activated protein kinases (MAPKs), such as ERK and p38 protein. The treatment of WAT preadipocytes with irisin did not result in their browning, but inhibited adipogenesis and effectively reduced the formation of new adipocytes [14,39]. The study of Pérez-Sotelo et al. on the C3H10T1/2 mesenchymal stem cell line confirmed that the lack of FNDC5 expression and secretion of irisin intensifies adipogenesis and reduces thermogenesis [40]. In addition, irisin enhances sensitivity to insulin by increasing glycogenesis and reducing gluconeogenesis [37]. Due to these properties, irisin may be an option to prevent and treat obesity and diabetes.

### 4.2. Irisin and Nervous System

Physical exercise has a beneficial effect on the functioning of the nervous system, especially on the hippocampus (the crucial structure for memory and learning), and results in better performance of cognitive functions [41]. Regular and moderate exercise increased the proliferation and differentiation of mouse neurons, increased their survival period, and stimulated migration [42]. Irisin plays an important role in the central nervous system. FNDC5 is expressed in Purkinje cells of the cerebellum [43] and in rodent hippocampus [44]. Moreover, the increase in FNDC5 expression correlates positively with the expression of brain-derived neurotrophic factor (BDNF), one of the most important signaling molecules for synaptic plasticity and neurogenesis in the hippocampus [44,45]. A lack of FNDC5 expression suppressed the differentiation of mouse embryonic stem cells into neurons and interfered with the maturation of astrocytes [46]. Moon et al. has also shown that irisin may regulate hippocampal neurogenesis in mice. Pharmacological doses of irisin (50–100 nM) increased the proliferation of mouse H19-7 hippocampal neuronal cells [47]. Irisin reduced oxidative stress-induced neuronal damage by inhibiting the secretion of proinflammatory cytokines, such as tumor necrosis factor α (TNFα) and IL-6, by the Akt/ERK1/2 signaling pathway in the mouse model of cerebral ischemia (MCAO) [48]. A positive correlation between serum irisin and BDNF levels, and cognition and episodic memory was observed after 10 weeks of physical training in adult volunteers at risk of dementia [49]. Exercises improve the mood, and more recently they were also considered to act as antidepressants. In addition to serotonin, irisin may also contribute to this effect, because decreased levels of this myokine is associated with mood swings. This effect of irisin is most likely associated with the activation of the PGC-1α/BDNF pathway [44,50]. Recent research showed that the levels of FNDC5/irisin in cerebrospinal fluid and hippocampi were reduced in patients with Alzheimer’s disease and the mouse model of this chronic neurodegenerative disease. The knockdown of FNDC5/irisin in mice brain cells impaired long-term potentiation and memory in the hippocampal region, while the expression of FNDC5/irisin restored hippocampal synaptic plasticity and memory in that animal model. Those recent findings revealed that irisin activity is important to synapse function and memory in a mouse model of Alzheimer’s disease and confirmed irisin-mediated beneficial effects of exercises on proper functioning of nervous system [51].

### 4.3. Irisin and Bones

Physical exercise is quite a strong incentive for stimulating bone formation. It has a beneficial effect on bone mineral density (BMD), increases the content of minerals, and reduces the risk of fractures, and hence regular exercise can prevent bone loss associated with the aging processes [52,53]. Colaianni et al. proved that irisin acts as a link between skeletal muscles and bones. This adipomyokine was found to exert a beneficial effect on cortical bone development in young mice treated with recombinant irisin for four weeks at a dose of 100 μg/kg/week. Irisin significantly increased the mass and strength of the cortical bones and positively modified their geometry by reducing the secretion of osteoblast inhibitors and causing an activation of activating transcription factor 4 (Atf4), and runt-related transcription factor 2 (Runx2), which consequently resulted in the expression of bone-specific genes; such as *Osx* (encoded an osterix) and *Col1a1* (encoded a collagen type I α1), and an increased activity of osteogenic cells [54]. It has also been shown in an in vitro study that irisin enhances the osteoblast differentiation process [55] through the activation of the aerobic glycolysis pathway [56]. It was suggested that the MAPK signaling pathway plays a key role in irisin-induced osteogenesis [54,55].

The results of clinical trials also indicate the positive effect of irisin on bone formation. Serbest et al. measured serum irisin concentration in patients with lower limb bone fractures who underwent an operation of bone fixation with closed intramedullary nailing. Irisin levels were determined on the day before surgery, also 1, 15, and 60 days after operation. A two-fold increase in serum concentration was observed 2 months after the operation compared to the concentration at other time points. The enhanced secretion of this myokine during bone healing process indicates its anabolic effects [57]. This result seems to be consistent with that reported by Anastasilakis et al. who showed that osteoporotic fractures in postmenopausal women have been associated with lower concentrations of irisin [58]. Irisin levels were lower in female athletes without menstruation in comparison to eumenorrheic athletes and non-athletes. In all athletes a positive correlation between irisin concentration and volumetric BMD and bone stiffness was observed [59]. The study by Palermo et al. confirmed the reduction of serum irisin concentration in donors with previous osteoporotic fractures. However, they reported no relationship between irisin levels and BMD [60].

Osteocytes in mature bone are found to be sensitive for irisin regulation. As mentioned in Section 3, this myokine acts on bone via integrin receptors, especially the members of αv integrin family. The physiological concentration of irisin (3–5 ng/mL) reduced the percentage of MLO-Y4 osteocyte-like cells undergoing apoptosis induced by hydrogen peroxide (H_2_O_2_). Besides showing the protective properties, irisin increased the expression of sclerostin (a specific glycoprotein, which acts as an inhibitor of bone formation and is responsible for bone remodeling) in MLO-Y4 osteocytes cultured in vitro and in an in vivo mouse model [28]. The stimulating effect of irisin on sclerostin expression observed by Kim et al. (2018), was opposite to the previous analysis of human serum samples using enzyme-linked immunosorbent assays (ELISA) which showed an inverse correlation between sclerostin and irisin levels [61].

The extensive research on the role of irisin in bone physiology underlines its important function in maintaining homeostasis between resorption and bone formation. The effects of irisin on osteocytes need further clarification.

### 4.4. Irisin in Inflammation

A lack of physical activity is an independent risk factor for the development of many chronic diseases, such as type 2 diabetes, obesity, immune dysfunction, asthma, and neurological or coronary heart disease. Most of those pathologies are associated with persistent, chronic inflammation. Regular and moderate physical activity positively affects the functioning of the immune system and reduces systemic inflammation [62]. Skeletal muscles modulate the inflammatory response mainly by the secretion of myokines [63], including irisin.

Our research demonstrated that regular physical training may alleviate the symptoms of inflammatory bowel diseases in obese mice, among others, by inhibiting the secretion of proinflammatory cytokines and increasing the amount of irisin released [64]. In vitro studies on RAW 264.7 macrophages showed that irisin regulates the activation of immunocompetent cells. It enhances the activity and proliferation of macrophages, improves their ability of phagocytosis, and reduces the production of reactive oxygen species (ROS) without affecting cell viability [65]. Irisin significantly reduces the extensive production of harmful H_2_O_2_ by macrophages. This antioxidative activity of irisin is a consequence of increased expression of key antioxidative enzymes, including superoxide dismutase (SOD), glutathione peroxidase (GSH-Px), and catalase 9 (Cat-9) [66]. The high concentrations of irisin (50 nM, 100 nM) reduced the number of both early and late apoptotic cells, attenuated the surface expression of Toll-like receptors 4 (TLR4), and diminished the amount of MyD88 adapter protein in lipopolysaccharide (LPS)-stimulated macrophages. The suppression of MAPK cascade signaling pathways and a consequential reduction in the levels of NF-κB nuclear transcription factor, c-Jun N-terminal kinases (JNK), and extracellular signal-regulated kinase (ERK) phosphorylation were also noticed. That led to a decrease in the release of key pro-inflammatory cytokines, such as IL-1β, IL-6, TNF-α, keratinocyte chemoattractant (KC), macrophage chemotactic protein (MCP)-1, and high mobility group box 1 (HMGB1) protein [67]. The results of these studies contribute to the understanding of the molecular mechanism underlying the anti-inflammatory effects caused by physical activity, and confirm the protective effect of irisin in the development of diseases associated with chronic inflammation.

### 4.5. Irisin in Obesity

Obesity is a state of excessive fat accumulation and is usually accompanied by insulin resistance, type 2 diabetes, cardiovascular diseases, and cancers [68]. The changes in the irisin levels were observed in the serum of obese people. Earlier studies reported the reduced concentration of irisin in obese individuals [25], while some of the recent studies showed the opposite results wherein the irisin levels were found to be increased in obese conditions [69,70]. Sahin-Efe et al. suggested a state of irisin resistance may be noticed during the course of obesity development (similar to leptin resistance) which could explain the elevated levels of irisin in these subjects [69]. According to Pardo et al., an increase of fat mass by 1 kg can lead to a twofold elevation of irisin level [71], while two other studies demonstrated that weight loss in obese subjects leads to a reduction of serum irisin levels [72,73].

Obesity is often related to the development of middle inflammation [68,74,75], also referred to as meta-inflammation, which is the state of chronic inflammation induced in non-immunocompetent tissues, such as muscles, intestines, adipose tissue, or liver, as a consequence of, e.g., the activation of resident macrophages [76]. Meta-inflammation may result in various metabolic abnormalities [77], and during the course of obesity, a massive influx of macrophages into fat tissue is observed [74,78].

Our recent study has shown that adipocytes are sensitive to irisin [79]. LPS-activated mature adipocytes mimic the state of obesity-related inflammation in vitro and when cultured in the presence of irisin, they produced and released significantly less amounts of pro-inflammatory cytokines, such as TNF-α, and IL-6. Irisin also decreased MCP-1 expression, thereby reducing chemotactic influx of macrophages. Furthermore, irisin impaired the expression and release of leptin, an adipokine associated with pro-inflammatory activation, and upregulated the level of anti-inflammatory cytokine adiponectin [79]. The elevated concentration of leptin in the serum of obese subjects contributes to excess food intake and reduced energy expenditure [80], and can result in the development of leptin resistance [81]. Leptin activity is closely associated with insulin resistance and the development of metabolic syndrome [82]. Gutierrez-Repiso et al. demonstrated that leptin plays a role in the expression of FNDC5 in adipose tissue [83]. Adiponectin, an anti-inflammatory adipokine, increases the sensitivity of the cell to insulin, whose level is found to be decreased in obesity, and may be associated with the development of insulin resistance [80]. Huo et al. demonstrated that irisin via heme oxygenase 1 (HO-1)/adiponectin axis improves perivascular adipose tissue (PVAT) function in diet-induced obese mice and attenuates the anti-contraction effect of PVAT [84]. Irisin improved endothelial function in obese subjects via activation of AMPK-eNOS pathway [85]. The protective effect of irisin was manifested by a reduction of TNF-α, an augmentation of adiponectin level, and an upregulation of lipid and glucose metabolism in mice fed on a high-fat diet [84]. Considering the positive anti-inflammatory effects of irisin on both adipocytes and macrophages, it seems reasonable to look for factors that increase sensitivity to irisin.

### 4.6. Irisin in Carcinogenesis

Irisin is also involved in carcinogenesis, although its role in cancer progression is currently ambiguous. It was found that higher concentrations of this adipomyokine in women are associated with a lower risk of breast cancer [86]. Women with primary breast cancer showed higher concentration of irisin in comparison to patients with spinal metastasis (*p* = 0.022) [87]. On the other hand, patients with renal cell cancer have higher levels of serum irisin (*p* = 0.0001) compared to the controls [88]. In vitro studies showed that irisin suppresses cell proliferation, migration, and viability of MCF-7 and MDA-MB-231 breast cancer malignant cell lines by stimulating caspase activity and inducing apoptosis, but do not affect MCF-10A normal breast epithelial cells. Moreover, the sensitivity of MDA-MB-231 malignant cells to the cytotoxic antineoplastic antibiotic doxorubicin (Dox) was increased in the presence of irisin, and it resulted in the higher breast cancer cytotoxicity [89]. Irisin also inhibited the growth, migration, and invasion of MIA PaCa-2 and Panc03.27 pancreatic cancer cells cultured in vitro via the AMPK-mTOR pathway signaling, by increased phosphorylation of AMP-activated protein kinase (AMPKα) and reduction of mammalian target of rapamycin (mTOR) phosphorylation [90]. A similar effect on cell proliferation and motility was also found in the case of U2OS and MG-63 osteosarcoma cell lines. An inversion of IL-6-induced epithelial–mesenchymal transition (EMT) and an inhibition of STAT3/Snail pathway signaling were observed in the presence of irisin [91]. The protective effect of irisin was also demonstrated for A549 and NCI-H446 lung cancer cells which exhibited suppressed EMT and invasion by decreasing the expression of Snail in the PI3K/Akt/Snail pathway [92]. The anti-cancer activity of irisin was not confirmed for human nor mouse colon, esophageal, thyroid and endometrial cell lines, which exhibited no change in the proliferation and adhesion properties in the presence of this myokine [93]. While cell proliferation, migration, and invasion of HepG2 and SMCC7721 hepatocellular cell lines were promoted by irisin, their sensitivity to Dox was reduced due to the activation of the Akt/PI3K pathway [94]. The above-mentioned discrepancy suggests that the action and role of irisin in the initiation, promotion, and progression of cancer may be tissue and cell specific.

## 5. Conclusions

The health-promoting effects of physical activity have always been known. The discovery of irisin in 2012 by Boström et al. revealed for the first time the role of this protein in causing the beneficial effects of exercise at a macromolecular level. Shortly after the discovery of irisin, numerous studies proved that this myokine is released into the circulation during physical exercise and that it is a multifunctional protein that regulates the biological functioning of various cells and tissues (Table 1). The activity and serum concentration of this myokine depends on the physiological and/or pathological state.

Most of the functions predicted for irisin require further studies to confirm or verify the previous results. First of all, the precise mechanism of irisin action has to be determined, from an interaction with the receptor, through a cascade of the intracellular signal transmission, to the direct effect on target cell metabolism, or with the release of biologically active factors. A recombinant non-glycosylated form of irisin has been used in most of the functional studies. As it was mentioned in Section 2, *N*-glycosylation of irisin is crucial to its secretion and the browning of adipocytes (Figure 2), and it seems possible that *N*-glycans also affect other activities of irisin. Future studies should be aimed at determining other glycosylation-dependent irisin functions and potential glycosylation changes that occur in pathological conditions, because numerous studies have demonstrated that glycosylation of different proteins is subject to change, mainly in carcinogenesis [95,96] and inflammation [97]. The detailed analysis of irisin glycosylation patterns and the effects of this post-translational modification on irisin actions are also crucial in the context of prophylactic and therapeutic uses of irisin.

## Figures and Tables

**Table 1:** Irisin activity and functions in selected physiological and pathological conditions.

Physiological/Pathological Conditions	Irisin Activity	References
Adipose (obesity)		
BAT	—Modulation of UCP-1 expression in mitochondria and enhancement of thermogenesis	[36,37,38]
	—Increase of energy consumption and metabolism of lipids and glucose	
WAT	—Increase of UCP-1 expression, leading to a conversion of the phenotype to BAT (browning)	[14,39]
Nervous system	—Induction of hippocampal neurogenesis by regulation of BDNF expression	[44,47]
	—Reduction of neuronal injury	[48]
Bones	—Increase of cortical bone mass and strength, enhancement of osteoblast differentiation process	[54,55]
	—Induction or inhibition of sclerostin expression	[28,61]
	—Increase in volumetric BMD or no relationship	[59,60]
Inflammation	—Enhancement of macrophage activity and proliferation, improvement of phagocytosis, and reduction of ROS production	[65]
	—Increase of the expression of antioxidative enzymes	[66]
	—Reduction of the release of proinflammatory cytokines	[67]
Carcinogenesis	—Suppression of proliferation, migration, and viability of breast, pancreatic, osteosarcoma and lung cancer cells	[89,90,91,92]
	—No effect on colon, esophageal, thyroid, and endometrial cancer cell progression	[93]
	—Promotion of proliferation, migration, and invasion of hepatocellular cancer cells	[94]
